# The Characteristics and Laboratory Findings of SARS-CoV-2 Infected Patients during the First Three COVID-19 Waves in Portugal—A Retrospective Single-Center Study

**DOI:** 10.3390/medicina60010059

**Published:** 2023-12-28

**Authors:** Cristiana P. Von Rekowski, Tiago A. H. Fonseca, Rúben Araújo, Carlos Brás-Geraldes, Cecília R. C. Calado, Luís Bento, Iola Pinto

**Affiliations:** 1 ISEL—Instituto Superior de Engenharia de Lisboa, Instituto Politécnico de Lisboa, Rua Conselheiro Emídio Navarro 1, 1959-007 Lisbon, Portugal; tiago.alexandre.hf@gmail.com (T.A.H.F.); rubenalexandredinisaraujo@gmail.com (R.A.); cecilia.calado@isel.pt (C.R.C.C.); 2 NMS—NOVA Medical School, FCM—Faculdade de Ciências Médicas, Universidade NOVA de Lisboa, Campo dos Mártires da Pátria 130, 1169-056 Lisbon, Portugal; luis.bento@chlc.min-saude.pt; 3 CHRC—Comprehensive Health Research Centre, Universidade NOVA de Lisboa, 1150-082 Lisbon, Portugal; 4 CEAUL—Centro de Estatística e Aplicações, Universidade de Lisboa, 1749-016 Lisbon, Portugal; 5 CIMOSM—Centro de Investigação em Modelação e Optimização de Sistemas Multifuncionais, ISEL—Instituto Superior de Engenharia de Lisboa, 1959-007 Lisbon, Portugal; 6 Intensive Care Department, CHULC—Centro Hospitalar Universitário de Lisboa Central, 1150-199 Lisbon, Portugal; 7 Integrated Pathophysiological Mechanisms, CHRC—Comprehensive Health Research Centre, NMS—NOVA Medical School, FCM—Faculdade de Ciências Médicas, Universidade NOVA de Lisboa, 1169-056 Lisbon, Portugal; 8 Department of Mathematics, ISEL—Instituto Superior de Engenharia de Lisboa, 1959-007 Lisbon, Portugal; iola.pinto@isel.pt; 9 NOVA Math—Center for Mathematics and Applications, NOVA SST—Nova School of Sciences and Tecnology, Universidade NOVA de Lisboa, 2829-516 Caparica, Portugal

**Keywords:** coronavirus disease 2019, intensive care unit, mortality, blood biomarkers, coronavirus disease 2019 waves

## Abstract

*Background and Objectives*: Given the wide spectrum of clinical and laboratory manifestations of the coronavirus disease 2019 (COVID-19), it is imperative to identify potential contributing factors to patients’ outcomes. However, a limited number of studies have assessed how the different waves affected the progression of the disease, more so in Portugal. Therefore, our main purpose was to study the clinical and laboratory patterns of COVID-19 in an unvaccinated population admitted to the intensive care unit, identifying characteristics associated with death, in each of the first three waves of the pandemic. *Materials and Methods*: This study included 337 COVID-19 patients admitted to the intensive care unit of a single-center hospital in Lisbon, Portugal, between March 2020 and March 2021. Comparisons were made between three COVID-19 waves, in the second (*n* = 325) and seventh (*n* = 216) days after admission, and between discharged and deceased patients. *Results*: Deceased patients were considerably older (*p* = 0.021) and needed greater ventilatory assistance (*p* = 0.023), especially in the first wave. Differences between discharged and deceased patients’ biomarkers were minimal in the first wave, on both analyzed days. In the second wave significant differences emerged in troponins, lactate dehydrogenase, procalcitonin, C-reactive protein, and white blood cell subpopulations, as well as platelet-to-lymphocyte and neutrophil-to-lymphocyte ratios (all *p* < 0.05). Furthermore, in the third wave, platelets and D-dimers were also significantly different between patients’ groups (all *p* < 0.05). From the second to the seventh days, troponins and lactate dehydrogenase showed significant decreases, mainly for discharged patients, while platelet counts increased (all *p* < 0.01). Lymphocytes significantly increased in discharged patients (all *p* < 0.05), while white blood cells rose in the second (all *p* < 0.001) and third (all *p* < 0.05) waves among deceased patients. *Conclusions*: This study yields insights into COVID-19 patients’ characteristics and mortality-associated biomarkers during Portugal’s first three COVID-19 waves, highlighting the importance of considering wave variations in future research due to potential significant outcome differences.

## 1. Introduction

Given the significant impact of the Coronavirus disease 2019 (COVID-19) pandemic since late December 2019, research into demographic and clinical data, as well as the identification of potential laboratory findings associated with the disease’s prognosis remains critical. The treatment of COVID-19 patients in the intensive care unit (ICU) involves managing underlying illnesses, managing preexisting comorbidities, and preventing ICU-related complications [1]. Despite all the new measures applied during and after the pandemic, numerous studies still report significant mortality in ICUs, highlighting the need for better indicators of the disease’s prognosis in critically ill patients [2,3,4,5].

Several biomarkers associated with cardiac damage, coagulation, and inflammation have been thoroughly examined, in order to identify patients at greater risk of death [6,7]. For example, myocardial ischemia/injury and myocarditis itself were considered as some of the leading causes of death in COVID-19 patients [8]. In a retrospective cohort study, more than half of the deceased patients had increased high-sensitivity cardiac troponin I (hs-cTn I) during hospitalization, which was associated with disease severity [9]. Dysfunction in the coagulation process has also been reported in COVID-19 patients, evidenced by high levels of D-dimers, fibrin degradation products, and prothrombin time, and by manifestations in other coagulation indexes [10,11]. Concerning inflammatory biomarkers, increased procalcitonin and C-reactive protein (CRP) levels have been linked to a higher risk of death and severe infection by severe acute respiratory syndrome coronavirus 2 (SARS-CoV-2) [12,13]. Like with any other viral infection, COVID-19 patients present with lymphopenia, which has been associated with a poor prognosis [3,14,15,16]. Decreases in eosinophil counts have also been reported several times and considered a severity biomarker [16,17]. A common feature in severe cases is elevated white blood cell (WBC) levels (leukocytosis), which, when supported by neutrophilia, lead to even less favorable outcomes [18,19]. Another indicator, recently used as a severity biomarker in COVID-19 cases, is the platelet-to-lymphocyte ratio (PLR), a relatively new and inexpensive marker of systemic inflammation [20,21]. According to Hashem et al. [22], in patients requiring ICU admission, PLR could be used as a predictor of severe infection by SARS-CoV-2. However, more evidence is needed in this matter, and so is a reliable cut-off value [23]. Neutrophil-to-lymphocyte ratio (NLR) is another easily calculated inflammatory biomarker. As opposed to PLR, NLR has revealed more reliable results and a high prognostic value [24].

As of November 2023, Omicron sub-lineages were predominantly responsible for most of the diagnosed COVID-19 cases in Portugal, with approximately seven cases of SARS-CoV-2 infection being detected per 100,000 habitants, each week. The disease’s specific mortality showed a stable trend, remaining below the threshold advocated by the European Centre for Disease Prevention and Control [25]. Nevertheless, over the course of the pandemic in Portugal, the all-age infection-fatality ratio for COVID-19 was one of the highest among 190 countries (2.085%, 95% uncertainty interval: 0.946–4.395) [26]. In spite of studies from all around the world exploring the potential of several biomarkers to predict adverse disease outcomes and mortality risk, in our understanding, few have analyzed the relevance of these biomarkers within distinct COVID-19 waves, more so in Portugal. As a result, we analyzed the laboratory findings, as well as the demographic and clinical data, of critically ill COVID-19 patients of a single-center hospital in Lisbon, Portugal, during the first three waves of the pandemic, a period in which most of the population was not vaccinated. It was important to exclude the effect of immunization in this study to have a better understating of the virus’s impact on this population as the pandemic evolved, facilitating the analysis of changes in severity over time. Hence, data were additionally compared between discharged and deceased patients, to identify specific disease characteristics associated with death in each of the waves.

## 2. Materials and Methods

### 2.1. Study Design and Sample

This retrospective observational study included unvaccinated patients diagnosed with COVID-19, with positive real-time reverse transcription–polymerase chain reaction assay test results, who were subsequently admitted to the ICU of the Centro Hospitalar Universitário Lisboa Central (CHULC) between March 2020 and March 2021.

**Figure 1:**
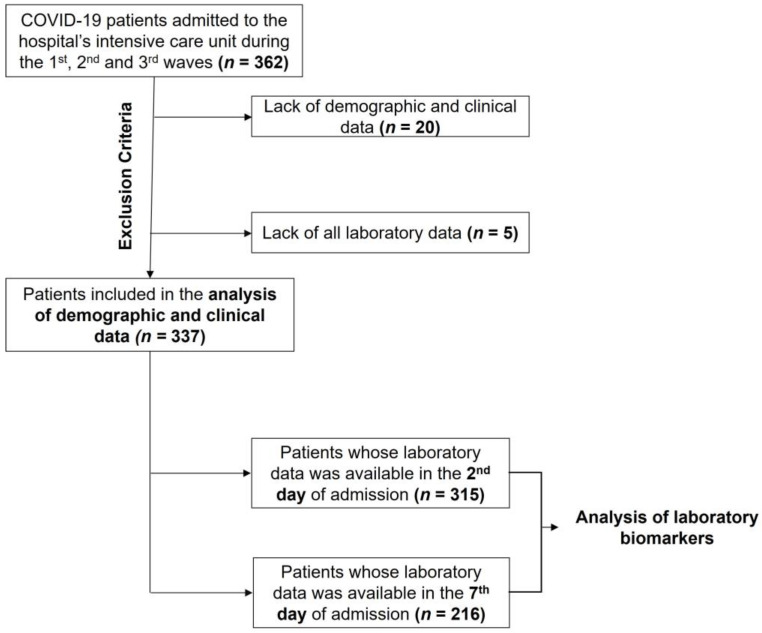
Flowchart for selecting patients in the present study.

Anonymized demographic, clinical and laboratory data from 362 patients was collected from the hospital’s electronic medical record system (Figure 1). Patients without hospital discharge, ICU admission and discharge dates (due to lack of data or because the individuals were still admitted) were excluded. From the remaining patients, those without laboratory results in the first week of ICU admission were also excluded. Finally, those over 18 years of age and with sufficient information regarding the study variables were included in the analysis of demographic and clinical data.

Since one of the goals of this study was to characterize the study sample on the first day of ICU admission, and most variables had several missing values on that specific day, the first results available in the first 48 h were used. As a result, 315 patients’ laboratory test results were analyzed from this time period. To assess the patients’ progress after one week in the ICU, the seventh day after admission was selected. The laboratory results of 216 patients were considered at this time.

### 2.2. Study Definitions

Regarding the COVID-19 waves in Portugal, the first one was considered to be from 10 March to 22 August 2020, the second was considered to be from 23 August to 19 December 2020, and the third was considered to be from 20 December 2020, to 5 June 2021. First, patients were grouped by wave according to their real-time reverse transcription–polymerase chain reaction assay diagnosis or symptom onset, whichever occurred earlier. Then, for each wave, patients were categorized according to their outcome (discharged from the ICU or deceased in the ICU).

Age and body mass index (BMI) were calculated with data collected at hospital admission. Several comorbidities were considered, although, because of the small frequencies, only some of them were reported, and others were grouped. Carcinomas, prostate adenocarcinomas and bladder, colon, breast, thyroid, prostate, and lung cancers were grouped as solid cancers. Regarding hematologic cancers, myeloproliferative syndrome, chronic lymphatic leukemia, multiple myeloma, and Hodgkin’s lymphoma were also clustered. Respiratory support integrated invasive mechanical ventilation (IMV), extracorporeal membrane oxygenation (ECMO), and high-flow oxygen (HFO). Outcome variables for all patients included the median number of days at the ICU, and at the hospital. The number of deaths in the hospital, after ICU discharge, was also calculated, and so were the median number of days between ICU and hospital discharge.

Daily maximum or minimum results were obtained for each of the selected laboratory variables in this study to obtain a single measure per patient, per day. Maximum levels of hs-cTn I, creatine kinase (CK), myoglobin, and lactate dehydrogenase (LDH) were considered cardiac biomarkers. Minimum platelet counts and maximum International Normalized Ratio (INR), D-dimers, fibrinogen, and ferritin levels were selected for coagulation biomarkers. Maximum levels of procalcitonin, CRP, WBC counts, neutrophil counts, and minimum eosinophil and lymphocyte counts were established as inflammatory biomarkers. Maximum PLR and NLR were calculated and considered inflammatory biomarkers.

Based on the hospital’s directives, the following categorical variables were generated regarding either the lower or the upper reference limits of the formerly mentioned variables: increased hs-cTn I (≥34.2 pg/mL), in risk of coronary syndrome (CS) (hs-cTn I ≥ 64.0 pg/mL), increased CK (≥200.0 U/L), increased myoglobin (≥85.0 ng/mL), increased LDH (≥220.0 U/L), increased INR (≥1.20), decreased platelets (<150 × 10^9^/L), increased D-dimers (≥230.0 µg/L), increased fibrinogen (≥4 g/L), increased ferritin (≥340 ng/mL), increased procalcitonin (≥0.06 ng/mL), increased CRP (≥5.0 mg/L), increased WBC counts (≥10.0 × 10^9^/L), increased neutrophil counts (≥7.0 × 10^9^/L), decreased eosinophil counts (<0.03 × 10^9^/L), and decreased lymphocyte counts (<0.8 × 10^9^/L). Two other variables were also obtained, namely increased PLR (≥192) and increased NLR (≥3.53), according to the reference limits respectively identified by Hashem et al. [22] and Forget et al. [27].

### 2.3. Statistical Analysis

Categorical data were presented as absolute frequencies and percentages. Continuous variables were expressed by their medians and interquartile range (25th percentile–75th percentile), since they were presented with an asymmetric distribution and deviations from normality. To assess the normality of the continuous variables, Kolmogorov–Smirnov and Shapiro–Wilk tests were used, as appropriate.

Concerning nominal variables, to make comparisons between two independent groups, namely, to compare discharged and deceased patients, non-parametric chi-square or Fisher’s exact tests (if the applicability conditions of the first test were not verified) were used. For continuous variables, the Mann–Whitney *U* test was applied. For comparisons between three or more independent groups, for example, to compare continuous variables between the three COVID-19 waves, the Kruskal–Wallis One-Way ANOVA test was used. Dunn’s post hoc tests were carried out on each pair of groups, using the Bonferroni adjustment in each Dunn’s *p*-value.

To infer the evolution of the patients’ condition in the first week of ICU admission, a comparative analysis of their laboratory results was conducted between the data from the second and the seventh days after ICU admission. The Wilcoxon matched-pairs signed-rank test was used to compare the results of continuous variables between the two mentioned days. The corresponding effect size (r) was calculated by means of the Matched-pairs rank biserial correlation coefficient, and interpreted according to Cohen’s classification of effect sizes which is 0.1 (small effect), 0.3 (moderate effect), and 0.5 and above (large effect) [28]. To visualize the biomarkers’ time course, median band lines plots were used.

To study the death outcome, event-free survival rates were obtained using the Kaplan–Meier estimator. To compare the survival curves, obtained according to cut-off points that separated the normal and non-normal results of a biomarker, the Log Rank (Mantel–Cox), Breslow (Generalized Wilcoxon) and Tarone–Ware tests, were used, as appropriate.

Descriptive and inferential statistics were obtained using IBM SPSS Statistics software, version 26 (IBM Corp., Armonk, NY, USA) and median band lines plots were generated using STATA software, version 12 (StataCorp. LLC, College Station, TX, USA). Statistical significance was set for two-sided *p* values of less than 0.05.

## 3. Results

### 3.1. Demographics and Clinical Characteristics during the COVID-19 Waves

From March 2020 to March 2021, a total of 337 COVID-19 patients met the study’s inclusion criteria; this included 62, 136, and 139 patients from the first, second, and third waves, respectively. Characteristics of all patients, as well as the comparisons between discharged and deceased ones, were detailed for each wave (Table 1).

The highest percentage of deaths in the ICU occurred in the second wave (39%), followed by the third and first waves (37% and 32%, respectively). Patients in the first wave were significantly older than those in the third (*p* = 0.022), and deceased patients had higher median ages compared to those who were discharged (*p* = 0.005, *p =* 0.001, and *p* < 0.001 for each wave, respectively). Arterial hypertension, the most common comorbidity, was found in at least 50% of patients in all waves, with significantly higher occurrences among deceased patients in the second and third waves.

The demand for IMV, ECMO, and HFO differed significantly across waves (*p* = 0.023, *p* < 0.001, and *p* = 0.009, respectively). Both IMV and ECMO were more frequently required in the first wave. Additionally, the need for IMV was significantly higher in deceased patients in all waves. However, time under IMV did not differ significantly between deceased and discharged patients. HFO was significantly more required in discharged patients, both in the second and third waves.

The ICU length of stay was similar between waves and did not differ between groups. For all discharged patients, the median hospital length of stay after ICU discharge was of ten days. After ICU discharge, five of those patients (8%) passed away in the first wave, seven (5%) in the second wave, and four (3%) in the third wave.

### 3.2. Biomarkers Results by Wave

**Figure 2:**
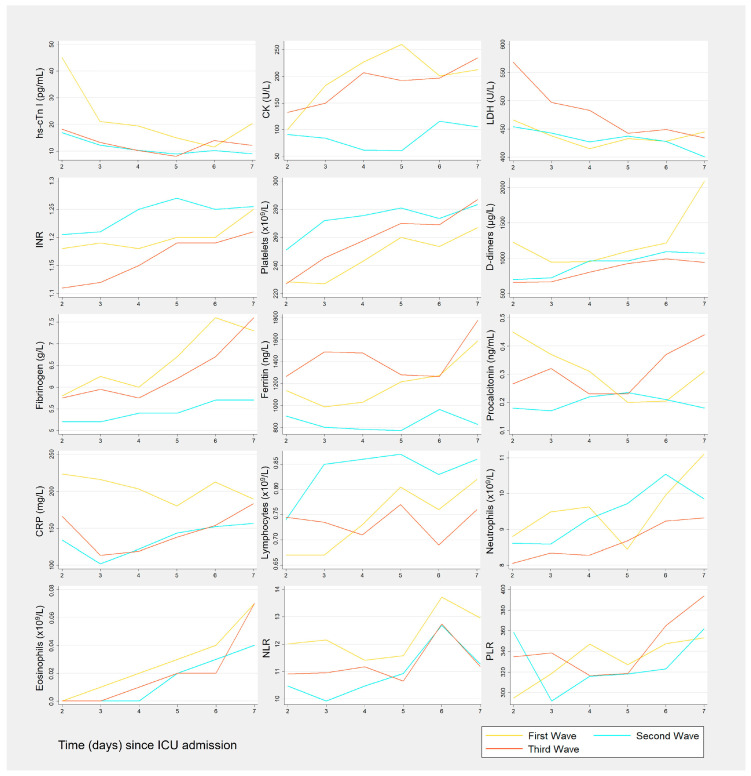
Serum biomarker time course, by wave, between the second and seventh days after ICU admission. hs-cTn I: high-sensitivity cardiac Troponin I; CK: creatine kinase; LDH: lactate dehydrogenase; INR: International Normalized Ratio; CRP: C-reactive protein; NLR: neutrophil-to-lymphocyte ratio; PLR: platelet-to-lymphocyte ratio.

The evolution of biomarkers in the first week of ICU admission, for each wave, is represented in Figure 2. The median results of biomarkers like hs-cTn I, CK, D-dimers, fibrinogen, CRP, eosinophils, and the NLR were higher in the first wave. INR, platelets, and lymphocyte median results were higher in the second wave, during the entire studied period. LDH and ferritin median results were higher in the third wave.

The biomarkers results were further compared between the three waves, in both the second and seventh days of ICU admission (Table 2 and Table 3, respectively). On the second day after ICU admission, median hs-cTn I, D-dimers, CRP, and eosinophil counts from the first wave were significantly higher than those from the second wave (*p* = 0.031, *p* = 0.045, *p* < 0.001 and *p* = 0.026, respectively), as obtained by pairwise comparisons between the results of the different waves. This could point to the more severe condition that patients from the first wave were in, compared to those from the second wave.

On both the second and seventh days, fibrinogen, ferritin, the INR and procalcitonin results were significantly different between waves (all *p* < 0.05). Compared to the second wave, procalcitonin levels on the second day were significantly higher in the first wave (*p* = 0.010), while on the seventh day these levels were higher in the third wave (*p* = 0.008). These findings highlight the impact of the different waves on the biomarkers’ behavior through time. In this specific case, procalcitonin levels tended to decrease over time in the first wave, and to increase in the third.

### 3.3. Biomarkers Results by Patient Outcome, for Each Wave

Considering the results obtained when comparing discharged and deceased patients, for both the second (Table 2) and the seventh (Table 3) days of ICU admission, the main findings by wave were described considering three different categories of laboratory biomarkers.

#### 3.3.1. Cardiac Biomarkers

Median hs-cTn I was solely above the established normality range on the second admission day, for both groups of patients in the first wave. In the remaining instances, hs-cTn I results were significantly higher for deceased patients (all *p* < 0.05). Considering CK and myoglobin, deceased patients consistently showed higher median concentrations, and a greater percentage of results outside normal ranges. On average, more than 95% of patients, in both the discharged and deceased groups, had elevated LDH levels across all of the waves.

**Figure 3:**
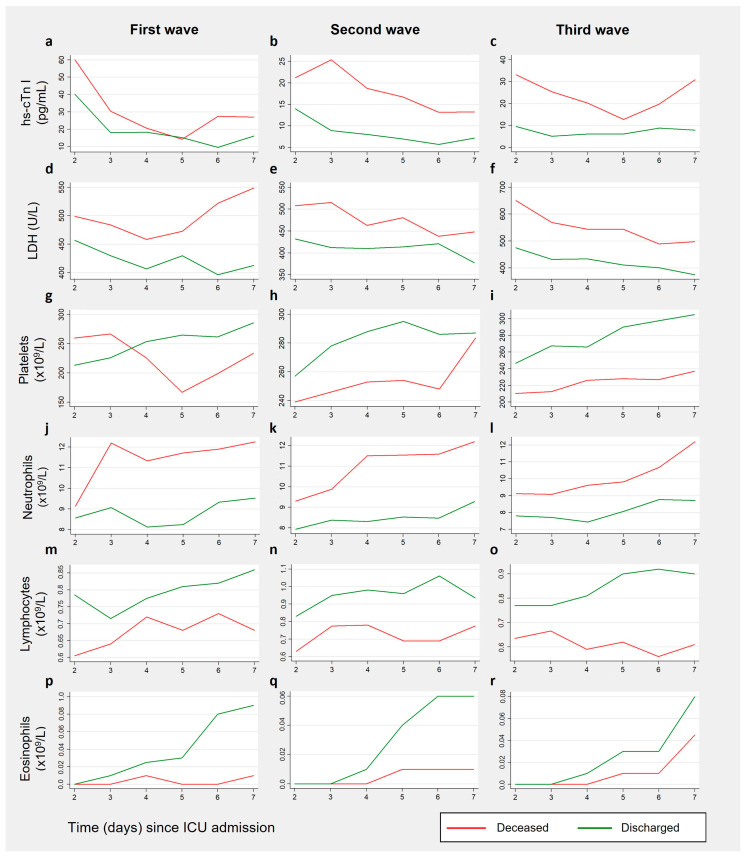
Time courses from serum hs-cTn I (**a**–**c**), LDH (**d**–**f**), platelets (**g**–**i**), neutrophils (**j**–**l**), lymphocytes (**m**–**o**), and eosinophils (**p**–**r**), by wave, for discharged/deceased patients during the first week of ICU admission. hs-cTn I: high-sensitivity cardiac Troponin I; LDH lactate dehydrogenase.

Independently of the wave, hs-cTn I level started with decreasing trends, having evidenced increases between the fifth and sixth days (Figure 3a–c). For discharged patients, in the first wave, the decrease of median hs-cTn I from the second to the seventh day was significant (*p* = 0.001; r = 0.751). The same was verified for both discharged (*p* = 0.001; r = 0.563) and deceased patients (*p* = 0.007; r = 0.506) in the second wave. (Table S1).

LDH levels reached their peak within the first two admission days and subsequently began to decline, except for deceased patients in the first wave (Figure 3d–f). For the remaining patients, considerable decreases between the results from the second and seventh days were observed across all waves (all *p <* 0.05, and r > 0.5) (Table S1).

#### 3.3.2. Coagulation Biomarkers

On the second admission day, median INR results were slightly above normal ranges for deceased patients from the second wave. However, on the seventh day, INR values consistently exceeded normal ranges for both groups of patients. The median platelet levels did not decrease below normality ranges in any case, but in the third wave, deceased patients exhibited significantly lower results, compared to discharged ones (*p* < 0.05 in both days). The maximum prevalence of thrombocytopenia was 20% for discharged patients and 25% for deceased ones. Median fibrinogen, D-dimer, and ferritin results were increased in both groups, across all waves.

Compared to the second day, the median INR results from all patients in the second and third waves increased on the seventh day (all *p* < 0.05). Platelet counts also increased for discharged patients (all *p* < 0.05), particularly in the first and third waves, while deceased patients showed lower results (Figure 3g–i). As observed in the previous section, median fibrinogen levels adopted upward trends, especially after the fourth day of ICU admission. The most noteworthy increases were observed for discharged patients from the first (*p* = 0.05, r = 0.845) and third (*p* = 0.008, r = 0.660) waves, and deceased patients from the third wave (*p* = 0.013, r = 0.790) (Table S1).

#### 3.3.3. Inflammation-Related Biomarkers

On both the second and the seventh days, procalcitonin and CRP median results were markedly raised, particularly in the first wave, and to a greater extent in the group of deceased patients. On the second day, at least half the patients of each group had lymphocytopenia, and more than 70% had eosinophilopenia. These percentages lowered on the seventh day. In all waves, median neutrophil counts, PLR, and NLR were above the established cutoff points.

Median WBC counts from deceased patients showed a significant rise in the second wave (*p* < 0.001, r = 0.611), and a less pronounced increase in the third wave (*p =* 0.024, r = 0.400). The same was verified for median neutrophil counts (*p* < 0.001, r = 0.614, for the second wave, and *p* = 0.047, r = 0.350, for the third wave) (Figure 3j–l). Median lymphocyte counts adopted increasing trends across all waves (Figure 3m–o), and moderate increases between the second and seventh days were observed for discharged patients (all *p* < 0.05, and r values between 0.3 and 0.5). Median eosinophil counts also significantly increased between these two days (all *p* ≤ 0.001, and r ranging from 0.6 to 0.8), except for deceased patients from the first wave. In the second and third waves, they remained null for up to four days for deceased patients, and for up to three days for discharged patients. Then, results started to fall within normal levels for discharged patients, whereas for deceased patients it only happened close to the seventh day of ICU admission (Figure 3p–r) (Table S1).

For each wave, an overview of all of the formerly mentioned biomarkers whose results were significantly different between the groups of discharged and deceased patients is provided in Table 4, for both the second and the seventh days after ICU admission.

### 3.4. Survival Analysis on the Second and Seventh Days after ICU Admission

**Figure 4:**
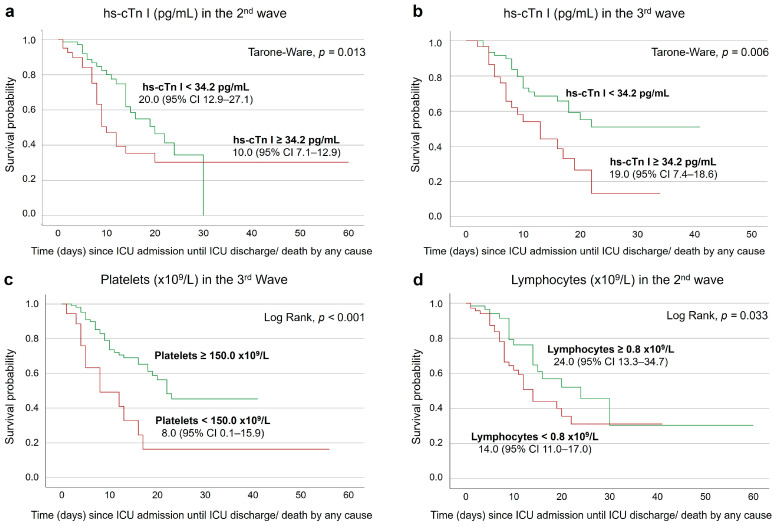
Event-free survival curves of COVID-19 patients, according to the hs-cTn I threshold of 34.2 pg/mL (**a**,**b**), platelet count threshold of 150.0 × 10^9^/L (**c**) and lymphocyte count threshold of 0.8 × 10^9^/L (**d**) on the second day after ICU admission, with median estimated survival time and respective 95% confidence interval (CI).

Kaplan–Meier survival analysis results, for the second day, demonstrated that the survival rate of the patients with hs-cTn I levels higher than 34.2 pg/mL was significantly lower in the second (*p* = 0.013) and third (*p* = 0.006) waves (Figure 4a,b). In the third wave, having platelet counts below 150.0 × 10^9^/L was associated with a lower survival rate (*p* < 0.001) (Figure 4c). The same was verified for patients whose lymphocyte counts were below 0.8 × 10^9^/L, on the second wave (*p* = 0.033) (Figure 4d).

**Figure 5:**
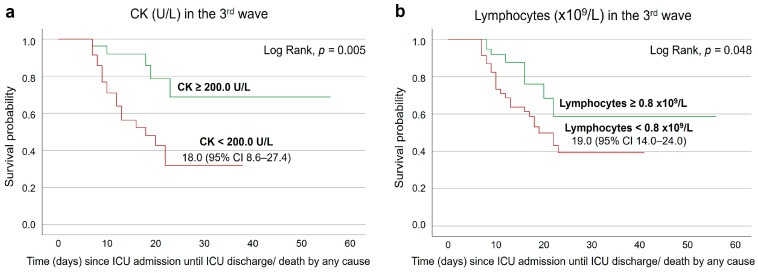
Event-free survival curves of COVID-19 patients, according to a CK threshold of 200.0 U/L (**a**), and lymphocyte count threshold of 0.8 × 10^9^/L (**b**) on the seventh day after ICU admission, with median estimated survival time and respective 95% CI.

On the seventh day after ICU admission, the survival rate of third-wave patients with CK results below 200.0 U/L was significantly lower (*p* = 0.005) than of those with results equal or above 200.0 U/L (Figure 5a). Still considering the third wave, having lymphocyte counts lower than 0.8 × 10^9^/L was associated with a lower survival rate (*p* = 0.048) (Figure 5b).

## 4. Discussion

Our study highlights the diverse demographic and clinical profiles of unvaccinated COVID-19 patients across the first three waves of the pandemic in Portugal. It also emphasizes the importance of considering these waves when evaluating patients’ laboratory biomarkers, given the observed variations across each wave. Notably, when data were compared between discharged and deceased patients, different sets of biomarkers associated with death were identified in each wave.

The majority of the study’s participants were enrolled in the second (40.4%) and third (41.2%) waves. This can be transposed to the general situation of the country since the majority of the cases were diagnosed in these two waves [29]. The spike Y839 variant is thought to have been responsible for the largest transmission chain in the country, being associated with 24.8% of the cases in the first 1.5 months [30,31]. At the beginning of the first COVID-19 wave in Portugal, most detected viruses included clades 20A (40%) and 20B (46%). Despite a sharp decline in intercontinental travel, summertime travel in Europe resumed, giving rise to new variants such as 20E (EU1) (B.1.177 lineage), reaching 70% of the cases in October [30,31]. The 20I (Alpha V1) variant (B.1.1.7 lineage) was first documented in the United Kingdom in September 2020, and rapidly overcame the 20E (EU1) variant, severely marking the third wave in Portugal, and reaching its peak frequency by April 2021 [32].

The patients’ median age throughout all waves was above 60 years old, however those from the first wave were significantly older and had a longer hospital stay. The same was concluded in a Spanish study involving five waves [33]. Additionally, patients in the first wave needed more IMV and ECMO, but for a shorter duration. Mortality did not significantly differ between waves, although being slightly higher in the second one. Comparably, in France, Contou et al. [34] observed that a smaller proportion of patients required IMV in the second wave, despite there being no differences regarding mortality between the first and second waves. Patients who died were considerably older across all waves, needed more IMV, and stayed longer in the hospital as compared to those who were discharged. In fact, older age was found to be a risk factor for IMV in COVID-19 patients, presumably leading to prolonged admission times, in addition to being an independent predictor of mortality [35,36].

Regarding laboratory biomarkers, most results in the first wave did not significantly differ between discharged and deceased patients, particularly on the second day. This may be due to the fact that both groups’ median results were proportionally outside of the established normalcy ranges. Therefore, one can conclude that patients from the first wave had similar laboratory findings, regardless of their outcome. In the second wave, for both the analyzed days, patients had differences regarding some of their cardiac and inflammation-related biomarkers. Lastly, in the third wave, biomarkers of all kinds were significantly different between patient groups, on both days, reflecting the higher disease severity of the patients who eventually died.

The median concentrations of hs-cTn I and the proportion of patients with elevated findings and/or at risk of CS were higher for deceased patients, across all waves over the two studied days. Despite being stated that those with cardiac damage are at a greater risk of death (hazard ratio 3.41 (95% CI; 1.62–7.16)) [37], other studies also suggest that besides myocardial injury being common, it is mostly associated with a low elevation of hs-cTn I at admission [38]. The current study came to comparable results. Even though the proportion of patients with raised hs-cTn I levels was significant on both days, the median values of this marker were, depending on the wave, only slightly elevated or within normality. More important than analyzing a single troponin measure, its variation over time is crucial. For instance, there are several cases where the levels of cardiac troponins from survivors remain stable, while the ones from non-survivors continue to rise until death [8,9,39]. In addition, in this study sample, health professionals observed cases of acute myocarditis and other complications that could have led to the elevation of cardiac troponins. Reports including such information weren’t accessible when the data were acquired, but it is critical to examine this type of information in the future. The hs-cTn I median levels were significantly higher in the first wave, which is consistent with other studies conducted in the same period. In the United Kingdom, researchers even found that there may be a link between a longer “symptom-to-call” time, higher peak hs-cTn I levels, and a lower six-month survival rate following myocardial infarction in COVID-19 patients [40]. Possible triggers were changes in patients’ behavior due to the pandemic, such as hospital/medical care avoidance to prevent contracting the disease [40,41]. Hence, this type of information can be valuable for health professionals when taking into consideration the results of patients’ cardiac biomarkers, not only in the present, but in the event of future incidents and new SARS-CoV-2 variants.

LDH median results in this study were above normal ranges in all waves, regardless of the patients’ outcome. LDH is significantly associated with disease severity, which might be connected to the typical multiorgan dysfunction that COVID-19 patients experience, which includes systemic inflammation, tissue destruction, and necrosis [42]. LDH levels peaked approximately in the first two days after ICU admission for all patients. Marked increases in this biomarker during the initial stages of the disease have been documented by other authors, who classified LDH as one of the most premature biomarkers related to inflammation and cell death in COVID-19 [43]. Regarding CK, deceased patients almost always had higher median concentrations and higher frequency of patients with values outside of normality ranges. Although elevated CK has been associated with increased mortality and disease severity, it is still unclear whether this elevation is due to the direct damage caused by the virus itself or by other factors, such as renal impairment, the toxic effects of cytokines, preexisting myopathies, and muscle dysfunction acquired during hospital stay/disease course [44,45,46].

D-dimers were higher for deceased patients in all waves, but significant differences between groups were only verified in the first and third waves. This biomarker can help diagnose thrombosis and can be used as an indicator for poor prognosis in the preliminary stages of infection [18]. The elevated results seen in this study, not only for deceased patients, can be related to the fact that D-dimers are higher in COVID-19 patients that need intensive care. According to Zhang et al. [47] levels above 2 mg/L can be used as mortality indicators with 92% sensitivity and 83% of specificity. Some patients also exhibited decreased platelet counts, an important finding since COVID-19 patients with thrombocytopenia have a threefold higher risk of death [48]. In the first wave, a higher percentage of patients with low platelet counts, and high D-dimers levels was observed. Once more, the fact that patients were told to stay at home and that the period until hospital admission may have been longer, can be one explanation for the first wave’s poorer results. In addition, other factors, such as changes in SARS-CoV-2 variants and treatment courses as the pandemic progressed, could be at play.

Regarding inflammatory-related biomarkers, deceased patients demonstrated significantly worse results, mainly in the second and third waves. CRP and procalcitonin levels were noticeably increased on both days and across all waves. For levels greater than or equal to 40 mg/L, CRP has been suggested as a reliable indicator of COVID-19 severity and risk of death [12]. Procalcitonin levels were associated with an approximately fivefold increased risk of severe infection by SARS-CoV-2 (odds ratio: 4.76; 95% CI: 2.74–8.29) [13] and secondary bacterial infections [49,50]. Other pathogen-related infections were not considered in this study, despite the fact that doing so is crucial in the future since these influence the severity of the disease and increase the risk of mortality [51]. In the case of median neutrophil counts, significant increases between the second and seventh days were identified in the second and third waves for deceased patients. These types of increases in the first days after ICU admission, which persist throughout the patients ICU stay, were already described by Chen et al. [52]. In their report, these changes were correlated with fatal outcomes. By examining the graphic representation of median band lines for both WBC and neutrophil counts, one can infer that leukocytosis was partly supported by neutrophilia. This finding has been linked to worse outcomes [19]. Despite being in the lower range of normality in the first days after ICU admission, lymphocyte levels of discharged patients showed an increasing tendency in all waves. Deceased patients, on the other hand, maintained lower levels in the first week of ICU internment, or those even declined, as seen in the third wave. Similar findings were already reported in other large longitudinal studies, which linked restored lymphocyte counts with better outcomes [52,53]. In the first four days after ICU admission, median eosinophil counts were null for deceased patients of the second and third waves. These results are consistent with recent research, which demonstrated that in recovered patients, eosinophil counts of zero occurred in a lower percentage (49%) than in deceased patients (78%) [54]. After these low results, eosinophil counts tended to increase in this study (except for the group of deceased patients from the first wave). Because eosinophils have beneficial functions such as controlling the inflammation generated by neutrophils [54], it is reasonable to deduce that the fact that the eosinophil counts of dying patients took longer to increase led to worse outcomes.

Despite all of the valuable information that was gathered, some limitations, like the small study sample for each COVID-19 wave, should be considered. This is a single-center study, and as such, the findings might not be generalizable to other settings. Thus, a larger sample size might increase the statistical power of the study’s findings. Regarding the clinical and demographical analysis, several pieces of information were not available, such as the severity of certain cardiac, liver, and renal disorders. Furthermore, the impact of other concomitant diseases/infections, the early use of antivirals, immunomodulating medication, vaccines, and other treatments should be considered in future research. In terms of laboratory data, not all biomarker results were available at all time points during the patients’ ICU stay. Thus, ensuring the availability of data throughout the entire admission in future studies could enhance the statistical power of the results obtained from the selected biomarkers. Longitudinal studies involving more recent COVID-19 waves and all of the abovementioned non-included data could provide even more accurate information in the future. Exploring the impact of immunization in this disease, particularly in the context of the more recent COVID-19 waves that featured different variants, could also be a noteworthy target for future research.

## 5. Conclusions

This study is, to the best of the authors’ knowledge, the largest in Portugal and one of the few that took into consideration COVID-19 waves in the period in which the population was not vaccinated. The present comparative analysis of patients’ demographics and clinical data, but also cardiac-, coagulation-, and inflammation-related biomarkers provides useful information for prognostic evaluation that can be used to guide treatment and monitoring, and a better understanding of the illness in the pre-vaccination waves. In the present, this is particularly important for patients that refused immunization. Besides, it also led to the conclusion that, due to several elements, each wave demonstrated very different behaviors (despite all biomarkers’ changes being comparable to reports from other countries). Information like this is crucial for future research, namely for the discovery of severity biomarkers and the development of predictive models for the disease’s outcomes, since not including the effect of its different variants/waves could give rise to misleading results. This was further confirmed by the distinct sets of biomarkers linked to death identified in each wave.

## Figures and Tables

**Table 1:** Demographics and clinical data from COVID-19 patients during the first three waves.

Features	First Wave (*n* = 62)			Second Wave (*n* = 136)			Third Wave (*n* = 139)			*p* Value ^a^
	Deceased		*p* Value	Deceased		*p* Value	Deceased		*p* Value	
	No (*n* = 42)	Yes (*n* = 20)		No (*n* = 83)	Yes (*n* = 53)		No (*n* = 88)	Yes (*n* = 51)		
Age										
Age, years	63 (50–74)	75 (68–79)	**0.005**	58 (47–70)	71 (63–79)	**<0.001**	56 (49–64)	69 (63–74)	**<0.001**	**0.021** ^b^
Sex										
Female	9 (21)	8 (40)	0.125	25 (30)	15 (28)	0.820	32 (36)	21 (41)	0.573	0.190
Male	33 (79)	12 (60)		58 (70)	38 (72)		56 (64)	30 (59)		
BMI										
BMI, Kg/m^2^	28 (24–31)	28 (26–31)	0.649	27 (25–29)	26 (24–28)	0.106	28 (25–31)	29 (26–33)	0.206	**0.013** ^c^
Comorbidities										
Arterial hypertension	23 (55)	11 (55)	0.986	38 (46)	41 (77)	**<0.001**	41(47)	38 (75)	**0.001**	0.912
Diabetes	15 (36)	7 (35)	0.956	30 (36)	23 (43)	0.398	27 (31)	23 (45)	0.088	0.840
Dyslipidemia	12 (29)	3 (15)	0.346	22 (27)	16 (30)	0.641	18 (21)	17 (33)	0.092	0.812
Obesity *	10/37 (27)	5/16 (31)	0.751	16/72 (22)	6/42 (14)	0.300	27/77 (35)	19/45 (42)	0.431	**0.008**
COPD	5 (12)	0 (0)	0.165	1 (1)	7 (13)	**0.006**	2 (2)	2 (4)	0.624	0.254
Myocardial ischemia	3 (7)	0 (0)	0.545	2 (2)	4 (8)	0.208	9 (10)	5 (10)	0.936	0.140
Chronic Kidney Disease	2 (5)	3 (15)	0.317	3 (4)	5 (9)	0.261	4 (5)	4 (8)	0.465	0.803
Solid Cancer	3 (7)	3 (15)	0.377	1 (1)	2 (4)	0.560	7 (8)	3 (6)	0.746	0.062
Hematologic Cancer	1 (2)	0 (0)	NR	2 (2)	0 (0)	NR	1 (1)	1 (2)	NR	NR
Respiratory Support										
IMV	34 (81)	20 (100)	0.046	47 (57)	47 (89)	**<0.001**	62 (71)	44 (86)	**0.035**	**0.023**
Days with IMV	7 (2–20)	6 (1–15)	0.398	6 (3–15)	7 (2–11)	0.928	8 (5–16)	8 (5–12)	0.583	0.091
ECMO	10 (24)	4 (20)	0.737	11 (13)	7 (13)	0.994	3 (3)	2 (4)	0.607	**<0.001**
Days with ECMO	8 (3–18)	8 (2–31)	NR	15 (7–31)	5 (3–15)	0.085	NR	NR	NR	0.791
HFO	13 (31)	4 (20)	0.366	45 (54)	10 (19)	**<0.001**	26 (30)	7 (14)	**0.035**	**0.009**
ICU and hospital length of stay										
Days in the ICU	8 (4–24)	11 (2–16)	0.751	8 (4–15)	8 (5–13)	0.719	9.0 (4–17)	8 (4–13)	0.329	0.619
Days in the hospital	27 (12–50)	16 (7–26)	**0.023**	20 (15–32)	11 (7–17)	**<0.001**	24 (13–40)	9 (5–14)	**<0.001**	0.062

Continuous variables are presented by their medians and interquartile range (25th percentile–75th percentile), and categorical variables by their absolute frequencies and percentages. *p* values for variables whose frequencies were too small/null were not reported (NR). ^a^ *p* values from comparisons of all patients’ data between waves. Significant *p* values from multiple comparisons following the Kruskal–Wallis One-Way ANOVA test, between ^b^ waves 1 and 3, and ^c^ waves 2 and 3 were denoted. Significant *p* values are highlighted in bold. COPD*:* Chronic obstructive pulmonary disease. * Due to missing values, the variable “Obesity” was reported as “obesity count/n (%)”.

**Table 2:** Comparisons between discharged and deceased patients’ biomarker results in the second day after ICU admission, for each wave.

Features	First Wave (*n* = 59)			Second Wave (*n* = 133)			Third Wave (*n* = 133)			*p* Value ^a^
	Deceased		*p* Value	Deceased		*p* Value	Deceased		*p* Value	
	No (*n* = 41)	Yes (*n* = 18)		No (*n* = 83)	Yes (*n* = 50)		No (*n* = 85)	Yes (*n* = 48)		
hs-cTn I (pg/mL)	40.1 (10.8; 248.0)	60.1 (26.2–213.0)	0.374	14.0 (4.5–45.3)	21.2 (8.2–275.7)	**0.042**	9.5 (4.8–39.2)	33.1 (15.1–147.7)	**0.003**	**0.029** ^b^
Increased hs-cTn I	21/39 (53.8%)	10/15 (66.7%)	0.393	19/69 (27.5%)	22/46 (47.8%)	**0.026**	14/55 (25.5%)	20/41 (48.8%)	**0.018**	**0.014**
CK (U/L)	113.5 (56.0; 388.5)	82.5 (31.8–209.0)	0.260	80.0 (34.0–170.0)	101.5 (53.8–275.8)	0.172	115.0 (42.0–342.0)	221.0 (60.0–393.0)	0.083	0.125
Increased CK	16/40 (40.0%)	4/18 (22.2%)	0.188	17/75 (22.7%)	13/36 (28.3%)	0.489	25/67 (37.3%)	23/43 (53.5%)	0.095	**0.010**
Myoglobin (ng/mL)	98.30 (32.3; 846.2)	143.4 (103.9–1150.2)	0.445	76.7 (42.8–398.7)	234.9 (58.9–721.8)	0.491	101.0 (68.3–226.1)	207.0 (74.8–260.5)	0.520	0.644
Increased Myoglobin	10 (62.5%)	5 (100%)	0.262	7 (41.2%)	5 (55.6%)	0.683	11 (73.3%)	11 (68.8%)	1.000	0.098
LDH (U/L)	457.0 (357.0–583.0)	499.0 (389.5–590.5)	0.593	432.0 (352.3–523.8)	508.0 (407.5–627.3)	**0.010**	475.0 (379.5–611.8)	650.5 (534.0–826.3)	**<0.001**	**<0.001** ^c,d^
Increased LDH	38/39 (97.4%)	17/17 (100%)	1.000	73/76 (96.1%)	43/44 (97.7%)	1.000	66/68 (97.1%)	44/44 (100.0%)	0.519	0.702
INR	1.2 (1.1–1.3)	1.2 (1.1–1.4)	0.857	1.2 (1.1–1.3)	1.2 (1.1–1.3)	0.455	1.1 (1.0–1.2)	1.1 (1.0–1.2)	0.429	**<0.001** ^c,d^
Increased INR	14/39 (35.9%)	7/18 (38.9%)	0.828	39/79 (49.4%)	24/47 (51.1%)	0.854	26/83 (31.3%)	9/48 (18.8%)	0.117	**0.001**
Platelets (×10^9^/L)	213.5 (166.0–270.0)	260.0 (156.8–337.5)	0.187	257.0 (196.0–308.0)	239.0 (191.0–297.0)	0.541	246.5 (198.8–326.5)	210.5 (147.0–266.8)	**0.006**	0.240
Decreased platelets	8/40 (20.0%)	3/18 (16.7%)	1.000	11/83 (13.3%)	5/47 (10.6%)	0.663	6/84 (7.1%)	12/48 (25.0%)	**0.004**	0.471
D-dimers (µg/L)	1127.0 (549.0–3679.0)	1305.0 (493.0–5997.8)	0.747	629.0 (330.0–2660.0)	853.0 (390.0–3652.0)	0.243	479.0 (268.5–1024.5)	795.0 (420.5–1922.5)	**0.024**	**0.002** ^b,c^
Increased D-dimers	36/37 (97.3%)	18/18 (100.0%)	1.000	68/79 (86.1%)	43/45 (95.6%)	0.131	60/73 (82.2%)	42/45 (93.3%)	0.086	0.057
Fibrinogen (g/L)	5.8 (3.8–7.5)	5.9 (4.9–7.5)	0.608	5.2 (4.4–6.5)	5.1 (3.8–6.1)	0.321	6.1 (5.0–7.0)	5.6 (4.7–6.8)	0.349	**0.041** ^d^
Increased fibrinogen	20/28 (71.4%)	14/16 (87.5%)	0.283	49/62 (79.0%)	26/36 (72.2%)	0.443	39/43 (90.7%)	27/29 (93.1%)	1.000	**0.028**
Ferritin (ng/mL)	1191.2 (731.7–2421.8)	947.6 (557.5–3173.3)	0.924	928.8 (584.5–1901.5)	770.3 (348.9–1798.0)	0.180	1265.6 (692.2–2508.2)	1271.0 (662.0–3639.8)	0.634	**0.032** ^d^
Increased ferritin	32/35 (91.4%)	14/15 (93.3%)	0.654	53/59 (89.8%)	29/34 (85.3%)	0.216	45/45 (93.8%)	23/26 (88.5%)	0.691	0.671
Procalcitonin (ng/mL)	0.4 (0.1–1.1)	0.7 (0.2–1.4)	0.299	0.1 (0.1–1.0)	0.3 (0.1–0.7)	0.238	0.2 (0.1–0.5)	0.6 (0.2–1.7)	**0.002**	**0.010** ^b^
Increased procalcitonin	32/32 (100.0%)	16/16 (100.0%)	NR	46/58 (79.3%)	36/41 (87.8%)	0.270	60/67 (89.6%)	41/43 (95.3%)	0.478	**0.004**
CRP (mg/L)	208.3 (117.5–254.3)	242.3 (176.1–283.1)	0.122	117.7 (63.5– 206.3)	148.1 (94.5– 225.3)	0.223	151.9 (67.4– 232.9)	199.9 (129.0–256.3)	**0.024**	**<0.001** ^b^
Increased CRP	41/41 (100.0%)	18/18 (100.0%)	NR	83/83 (100.0%)	47/47 (100.0%)	NR	84/84 (100.0%)	48/48 (100.0%)	NR	NR
WBC (×10^9^/L)	10.7 (8.6–13.1)	10.8 (9.3–17.3)	0.290	9.6 (7.2–13.9)	10.8 (7.8–13.6)	0.453	9.0 (7.3–13.6)	10.0 (7.4–13.7)	0.488	0.254
Increased WBC	18/40 (45.0%)	9/18 (50.0%)	0.724	32/83 (38.6%)	23/47 (48.9%)	0.250	32/84 (38.1%)	23/48 (47.9%)	0.271	0.166
Lymphocytes (×10^9^/L)	0.8 (0.5–1.2)	0.6 (0.5–1.1)	0.501	0.8 (0.6–1.2)	0.6 (0.4–1.0)	**0.014**	0.8 (0.6–1.1)	0.6 (0.5–0.9)	**0.019**	0.900
Decreased lymphocytes	25/40 (62.5%)	12/18 (66.7%)	0.760	47/83 (56.6%)	33/47 (70.2%)	0.126	55/84 (65.5%)	33/48 (68.8%)	0.701	0.578
Neutrophils (×10^9^/L)	8.6 (6.7–11.3)	9.1 (7.9–14.1)	0.243	7.9 (5.3–12.5)	9.3 (6.9–12.4)	0.323	7.8 (5.8–11.6)	9.1 (6.4–12.5)	0.289	0.534
Increased neutrophils	20/40 (50.0%)	12/18 (66.7%)	0.238	39/83 (47.0%)	27/47 (57.4%)	0.252	38/84 (45.2%)	25/48 (52.1%)	0.449	0.112
Eosinophils (×10^9^/L)	0.00 (0.00–0.01)	0.00 (0.00–0.04)	0.915	0.00 (0.00–0.01)	0.00 (0.00–0.00)	**0.022**	0.00 (0.00–0.01)	0.00 (0.00–0.00)	**0.011**	**0.032** ^b^
Decreased eosinophils	32/40 (80.0%)	13/18 (72.2%)	0.516	73/83 (88.0%)	44/47 (93.6%)	0.374	68/84 (81.0%)	45/48 (93.5%)	**0.044**	0.077
PLR	256.1 (193.9–414.6)	435.3 (250.1–681.5)	0.064	330.6 (235.6– 429.1)	431.4 (269.8– 576.5)	**0.008**	324.7 (236.1–461.1)	365.7 (203.3–581.3)	0.601	0.424
Increased PLR	31/40 (77.5%)	14/18 (77.8%)	1.000	67/83 (80.7%)	43/47 (91.5%)	0.102	68/84 (81.0%)	38/48 (79.2%)	0.804	0.460
NLR	10.7 (5.5–15.8)	14.7 (9.2–29.0)	0.122	8.9 (5.6–15.5)	15.9 (8.9–23.5)	**0.003**	9.9 (6.7–15.3)	12.7 (8.5–17.8)	**0.032**	0.924
Increased NLR	37/40 (92.5%)	16/18 (88.9%)	0.641	79/83 (95.2%)	45/47 (95.7%)	1.000	81/84 (96.4%)	47/48 (97.9%)	1.000	0.244

Continuous variables were presented by their medians and interquartile range (25th percentile–75th percentile). Categorical variables were presented as absolute frequencies, followed by the number of patients with available data and percentages. Chi-square or Fisher’s exact tests were used for comparing categorical variables, and the Mann–Whitney U test was used for comparing two continuous variables. ^a^ *p* values using the Kruskal–Wallis One-Way ANOVA test for comparisons of all patients’ data between the three waves. Significant *p* values are highlighted in bold. Significant *p* values from multiple comparisons following the Kruskal–Wallis One-Way ANOVA test, between ^b^ waves 1 and 2, ^c^ waves 1 and 3, and ^d^ waves 2 and 3 were denoted. NR—not reported, *p* values for variables whose frequencies were too small/null.

**Table 3:** Comparisons between discharged and deceased patients’ biomarker results in the seventh day after ICU admission, for each wave.

Features	First Wave (*n* = 39)			Second Wave (*n* = 87)			Third Wave (*n* = 90)			*p* Value ^a^
	Deceased		*p* Value	Deceased		*p* Value	Deceased		*p* Value	
	No (*n* = 27)	Yes (*n* = 12)		No (*n* = 52)	Yes (*n* = 35)		No (*n* = 58)	Yes (*n* = 32)		
hs-cTn I (pg/mL)	16.1 (8.9–33.0)	27.0 (12.5–118.5)	**0.133**	7.2 (3.0–23.6)	13.3 (7.2–37.4)	**0.038**	7.9 (3.6–22.8)	30.8 (11.7–145.6)	**0.045**	0.061
Increased hs-cTn I	5/21 (23.8%)	5/11 (45.5%)	0.252	7/37 (18.9%)	7/28 (25.0%)	0.555	6/29 (20.7%)	9/19 (47.4%)	0.051	0.423
CK (U/L)	220.0 (55.0–479.5)	195.0 (69.0–373.0)	0.919	103.5 (20.8–331.5)	105.5 (50.3–243.5)	0.371	186.0 (71.0–483.0)	459.5 (206.0–849.8)	**0.011**	**0.008** ^d^
Increased CK	14/25 (56.0%)	5/11 (45.5%)	0.559	13/38 (34.2%)	9/26 (34.6%)	0.973	17/39 (43.6%)	19/24 (79.2%)	**0.006**	**0.040**
LDH (U/L)	413.0 (336.0–470.5)	548.5 (392.0–631.8)	**0.013**	377.5 (323.0–515.0)	448.0 (333.8–564.5)	0.194	374.5 (312.5–501.5)	498.0 (410.3–561.0)	**0.005**	0.557
Increased LDH	25/25 (100.0%)	12/12 (100.0%)	NR	46/48 (95.8%)	29/30 (96.7%)	1.000	48/40 (100.0%)	30/30 (100.0%)	NR	NR
INR	1.3 (1.2–1.4)	1.2 (1.1–1.3)	0.505	1.2 (1.2–1.3)	1.3 (1.2–1.4)	0.105	1.2 (1.1–1.3)	1.2 (1.1–1.3)	0.216	**0.012** ^d^
Increased INR	16/26 (61.5%)	6/12 (50.0%)	0.503	31/52 (59.6%)	26/34 (76.5%)	0.106	25/57 (43.9%)	21/32 (65.6%)	0.049	0.146
Platelets (×10^9^/L)	286.0 (222.0–407.0)	234.0 (178.3–296.3)	0.168	287.0 (223.0–358.8)	283.5 (212.3–373.5)	0.856	305.0 (237.5–385.5)	237.0 (184.5–316.5)	**0.005**	0.858
Decreased platelets	3/27 (11.1%)	2/12 (16.7%)	NR	4/52 (7.7%)	3/34 (8.8%)	NR	2/57 (3.5%)	5/32 (15.6%)	NR	0.631
D-dimers (µg/L)	1340.0 (435.0–2812.0)	3888.0 (1542.3–8718.8)	**0.018**	1052.0 (481.0–2473.0)	1693.0 (706.0–3175.0)	0.465	715.0 (479.5–1300.5)	1817.5 (941.0–2977.5)	**<0.001**	0.059
Increased D-dimers	27/27 (100.0%)	12/12 (100.0%)	NR	45/47 (95.7%)	30/31 (96.8%)	1.000	46/49 (93.9%)	30/30 (100.0%)	0.284	NR
Fibrinogen (g/L)	7.3 (6.5–9.7)	6.8 (3.7–8.4)	0.210	5.5 (4.8–7.2)	6.0 (4.4–8.2)	0.593	7.4 (5.2–9.4)	7.7 (5.8–9.3)	0.729	**0.004** ^d^
Increased Fibrinogen	12/13 (92.3%)	6/8 (75.0%)	0.531	23/26 (88.5%)	15/17 (88.2%)	1.000	19/19 (100.0%)	18/19 (94.7%)	1.000	NR
Ferritin (ng/mL)	1592.3 (837.8–2053.8)	1450.6 (1134.5–2325.7)	0.685	776.9 (359.8–1993.5)	935.2 (249.5–2722.6)	0.949	1330.2 (640.2–2431.2)	2073.0 (1283.2–3610.0)	0.057	**0.010** ^d^
Increased ferritin	21/22 (95.5%)	9/9 (100.0%)	1.000	20/26 (76.9%)	15/21 (71.4%)	0.668	23/24 (95.8%)	17/18 (94.4%)	1.000	**0.002**
Procalcitonin (ng/mL)	0.2 (0.1–1.1)	0.4 (0.2–1.9)	0.313	0.2 (0.1–0.5)	0.3 (0.1–0.8)	0.126	0.4 (0.1–0.8)	1.2 (0.3–5.9)	**0.008**	**0.010** ^d^
Increased procalcitonin	22/22 (100.0%)	9/9 (100.0%)	NR	21/31 (67.7%)	24/26 (92.3%)	**0.023**	40/42 (95.2%)	27/27 (100.0%)	0.517	**<0.001**
CRP (mg/L)	189.8 (83.6–258.1)	154.4 (52.3–260.7)	0.499	104.6 (35.9–226.0)	219.0 (119.6–267.9)	**0.003**	169.2 (74.5–260.7)	205.6 (154.5–283.5)	**0.019**	0.111
Increased CRP	27/27 (100.0%)	12/12 (100.0%)	NR	51/52 (98.1%)	34/34 (100%)	1.000	56/57 (98.2%)	32/32 (100.0%)	1.000	NR
WBC (×10^9^/L)	12.8 (8.6–14.2)	13.5 (10.7–19.7)	0.159	10.8 (7.9–14.3)	14.2 (10.6–18.4)	**0.014**	10.3 (8.9–13.2)	13.5 (9.5–18.8)	**0.043**	0.558
Increased WBC	17/27 (63.0%)	10/12 (83.3%)	0.276	32/52 (61.5%)	26/34 (76.5%)	0.149	31/57 (54.4%)	22/32 (68.8%)	**0.185**	0.436
Lymphocytes (×10^9^/L)	0.9 (0.7–1.4)	0.7 (0.5–1.3)	0.298	0.9 (0.6–1.3)	0.8 (0.4–1.0)	0.090	0.9 (0.6–1.4)	0.6 (0.4–0.8)	**0.002**	0.668
Decreased lymphocytes	10/27 (37.0%)	8/12 (66.7%)	0.087	20/52 (38.5%)	18/34 (52.9%)	0.186	23/57 (40.4%)	24/32 (75.0%)	**0.002**	0.502
Neutrophils (×10^9^/L)	9.5 (6.7–12.6)	12.3 (9.3–16.6)	0.104	9.3 (6.2–12.3)	12.2 (9.0–16.1)	**0.003**	8.7 (7.1–11.0)	12.2 (8.3–17.2)	**0.010**	0.694
Increased neutrophils	19/27 (70.4%)	11/12 (91.7%)	0.228	35/52 (67.3%)	31/34 (91.2%)	**0.010**	44/57 (77.2%)	26/32 (81.3%)	0.654	0.950
Eosinophils (×10^9^/L)	0.09 (0.01–0.15)	0.01 (0.00–0.11)	0.118	0.06 (0.01–0.13)	0.01 (0.00–0.05)	**0.004**	0.08 (0.02–0.19)	0.05 (0.01–0.13)	0.229	0.207
Decreased eosinophils	7/27 (25.9%)	7/12 (58.3%)	0.075	16/52 (30.8%)	20/34 (58.8%)	**0.010**	15/57 (26.3%)	13/32 (40.6%)	0.163	0.359
PLR	353.0 (190.9–500.0)	374.3 (200.2–508.8)	0.869	346.7 (201.2–527.8)	386.3 (248.9–526.3)	0.318	341.7 (200.0–512.9)	424.0 (304.2–590.4)	0.207	0.610
Increased PLR	20/27 (74.1%)	9/12 (75.0%)	1.000	40/52 (76.9%)	30/34 (88.2%)	0.187	46/57 (80.7%)	28/32 (87.5%)	0.411	0.501
NLR	8.8 (5.8–16.0)	17.8 (12.8–18.8)	0.056	9.3 (4.9–18.4)	14.1 (8.4–32.2)	**0.002**	9.6 (6.1–15.7)	15.4 (10.1–32.4)	**<0.001**	0.964
Increased NLR	26/27 (96.3%)	11/12 (91.7%)	0.526	49/52 (94.2%)	34/34 (100.0%)	0.274	56/57 (98.2%)	32/32 (100.0%)	1.000	NR

Continuous variables were presented by their medians and interquartile range (25th percentile–75th percentile). Categorical variables were presented as absolute frequencies, followed by the number of patients with available data and percentages. Chi-square or Fisher’s exact tests were used for comparing categorical variables, and the Mann–Whitney U test was used for comparing two continuous variables. ^a^ *p* values using the Kruskal–Wallis One-Way ANOVA test for comparisons of all patients’ data between the three waves. Significant *p* values are highlighted in bold. Significant *p* values from multiple comparisons following the Kruskal–Wallis One-Way ANOVA test, between ^d^ waves 2 and 3 were denoted. NR—not reported, *p* values for variables whose frequencies were too small/null.

**Table 4:** Overview of the biomarkers results that were significantly different between discharged and deceased patients.

	First Wave		Second Wave		Third Wave	
	2nd Day	7th Day	2nd Day	7th Day	2nd Day	7th Day
Cardiac biomarkers	ns	In risk of CS LDH (U/L)	hs-cTn I (pg/mL) ⬆ hs-cTn I LDH (U/L)	hs-cTn I (pg/mL)	hs-cTn I (pg/mL) ⬆ hs-cTn I LDH (U/L)	hs-cTn I (pg/mL) LDH (U/L) CK ⬆ CK
Coagulation biomarkers	ns	D-dimers (µg/L)	ns	ns	Platelets (×10^9^/L) ⬇ Platelets D-dimers (µg/L)	Platelets (×10^9^/L) D-dimers (µg/L)
Inflammation Biomarkers	ns	ns	Lymphocytes (×10^9^/L) Eosinophils (×10^9^/L) PLR NLR	⬆ Procalcitonin CRP (mg/L) WBCs (×10^9^/L) Neutrophils (×10^9^/L) ⬆ Neutrophils Eosinophils (×10^9^/L) ⬇ Eosinophils NLR	Procalcitonin (ng/mL) CRP (mg/L) Lymphocytes (×10^9^/L) Eosinophils (×10^9^/L) ⬇ Eosinophils NLR	Procalcitonin (ng/mL) CRP (mg/L) WBCs (×10^9^/L) Lymphocytes (×10^9^/L) ⬇ Lymphocytes Neutrophils (×10^9^/L) NLR

ns: not significant; hs-cTn I: high-sensitivity cardiac Troponin I; LDH: lactate dehydrogenase; CK: creatine kinase; PLR: platelet-to-lymphocyte ratio; NLR neutrophil-to-lymphocyte ratio; CRP C-reactive protein; WBC white blood cell. ⬆ Increased results; ⬇ Decreased results.

## Data Availability

The datasets used and/or analyzed during the current study are available from the corresponding author on reasonable request.

## Supplementary Materials

The following supporting information can be downloaded at: https://www.mdpi.com/article/10.3390/medicina60010059/s1. Table S1. Comparisons between median biomarkers’ levels on the second and seventh days after ICU admission for both groups of patients, for each wave.

## Abbreviations

COVID-19: coronavirus disease 2019; ICU: intensive care unit; hs-cTn I: high-sensitivity cardiac troponin I; CRP: C-reactive protein; SARS-CoV-2: severe acute respiratory syndrome coronavirus 2; WBC: white blood cell; PLR: platelet-to-lymphocyte; NLR: neutrophil-to-lymphocyte ratio; CHULC: Centro Hospitalar Universitário Lisboa Central; BMI: body mass index; IMV: invasive mechanical ventilation; ECMO: extracorporeal membrane oxygenation; HFO: high-flow oxygen; CK: creatine kinase; LDH: lactate dehydrogenase; INR: International Normalized Ratio; CS: coronary syndrome; NR: not reported; COPD: chronic obstructive pulmonary disease; NS: not significant; CI: confidence interval.
