# Supplementary material for: The Characteristics and Laboratory Findings of SARS-CoV-2 Infected Patients during the First Three COVID-19 Waves in Portugal—A Retrospective Single-Center Study
Source: Medicina (Kaunas). 2023 Dec 28;60(1):59. doi: 10.3390/medicina60010059 (PMC10817678; doi:10.3390/medicina60010059)
Supplement: Supplementary file 1 [file medicina-60-00059-s001.zip › medicina-2763413-supplementary.pdf]

Supplementary Table S1. Comparisons between median biomarkers' levels on the second and seventh days after ICU admission for both groups of patients, for each wave.

	**First Wave**				**Second Wave**				**Third Wave**			
	Deceased				Deceased				Deceased			
	No		Yes		No		Yes		No		Yes	
	p value	Effect size	p value	Effect size	p value	Effect size	p value	Effect size	p value	Effect size	p value	Effect size
hs-cTn I (pg/mL)	**<0.001**	0.751	0.374	0.296	**0.001**	0.563	**0.007**	0.506	0.081	0.356	0.076	0.431
LDH (U/L)	**0.007**	0.558	0.722	0.107	**<0.001**	0.589	**0.022**	0.439	**<0.001**	0.557	**<0.001**	0.805
INR	0.798	0.051	0.754	0.091	**0.004**	0.412	**0.025**	0.384	**<0.001**	0.453	**<0.001**	0.691
Platelets ($x10^9$/L)	**0.003**	0.591	1.000	0.000	**0.003**	0.409	0.108	0.276	**0.002**	0.405	0.256	0.201
D-dimers (µg/L)	0.276	0.218	0.530	0.181	0.196	0.193	0.926	0.017	0.928	0.014	**0.019**	0.443
Fibrinogen (g/L)	**0.005**	0.845	0.735	0.128	0.305	0.230	0.066	0.613	**0.008**	0.660	**0.013**	0.790
WBCs ($x10^9$/L)	0.091	0.331	0.136	0.430	0.672	0.059	**<0.001**	0.611	0.221	0.162	**0.024**	0.400
Lymphocytes ($x10^9$/L)	**0.005**	0.550	0.367	0.261	**0.021**	0.321	0.874	0.027	**<0.001**	0.460	0.594	0.094
Neutrophils ($x10^9$/L)	0.269	0.217	0.117	0.453	0.816	0.032	**<0.001**	0.614	0.636	0.063	**0.047**	0.350
Eosinophils ($x10^9$/L)	**0.001**	0.641	0.123	0.445	**<0.001**	0.746	**0.001**	0.585	**<0.001**	0.629	**<0.001**	0.780
NLR	0.469	0.142	0.308	0.294	0.489	0.096	**0.023**	0.388	**0.040**	0.272	0.112	0.281

Related-samples Wilcoxon matched-pair signed rank test *p* values for the comparison of the median biomarker values on the second day with those of the seventh day after ICU admission, for each group of patients, independently, in each of the waves. Only biomarkers with significant results in at least one group of one of the waves were presented. hs-cTn I – High-sensitivity cardiac troponin I; LDH – Lactate dehydrogenase; INR – International Normalized Ratio; WBC – White blood cell; NLR - Neutrophil-to-lymphocyte ratio.
